# Natural language word embeddings as a glimpse into healthcare language and associated mortality surrounding end of life

**DOI:** 10.1136/bmjhci-2021-100464

**Published:** 2021-10-27

**Authors:** Ivan Shun Lau, Zeljko Kraljevic, Mohammad Al-Agil, Shelley Charing, Alan Quarterman, Harold Parkes, Victoria Metaxa, Katherine Sleeman, Wei Gao, Richard J B Dobson, James T Teo, Phil Hopkins

**Affiliations:** 1 Kings College Hospital, King’s College Hospital NHS Foundation Trust, London, UK; 2 Department of Biostatistics and Health Informatics, King’s College London, London, UK; 3 Patients, (Private Individuals), London, UK; 4 School of Medical Education, King’s College London, London, UK; 5 Department of Palliative Care, Policy and Rehabilitation, King’s College London, London, UK; 6 Department of Biostatistics and Health Informatics, Institute of Psychiatry, Psychology and Neuroscience, King’s College London, London, UK; 7 Institute of Psychiatry Psychology and Neuroscience, King’s College London, London, UK; 8 Intensive Care Medicine, Anaesthesia and Trauma, King’s College Hospital NHS Foundation Trust, London, UK

**Keywords:** data science, documentation, electronic health records, medical records, patient-centered care

## Abstract

**Objectives:**

To clarify real-world linguistic nuances around dying in hospital as well as inaccuracy in individual-level prognostication to support advance care planning and personalised discussions on limitation of life sustaining treatment (LST).

**Design:**

Retrospective cross-sectional study of real-world clinical data.

**Setting:**

Secondary care, urban and suburban teaching hospitals.

**Participants:**

All inpatients in 12-month period from 1 October 2018 to 30 September 2019.

**Methods:**

Using unsupervised natural language processing, word embedding in latent space was used to generate phrase clusters with most similar semantic embeddings to ‘Ceiling of Treatment’ and their prognostication value.

**Results:**

Word embeddings with most similarity to ‘Ceiling of Treatment’ clustered around phrases describing end-of-life care, ceiling of care and LST discussions. The phrases have differing prognostic profile with the highest 7-day mortality in the phrases most explicitly referring to end of life—‘Withdrawal of care’ (56.7%), ‘terminal care/end of life care’ (57.5%) and ‘un-survivable’ (57.6%).

**Conclusion:**

Vocabulary used at end-of-life discussions are diverse and has a range of associations to 7-day mortality. This highlights the importance of correct application of terminology during LST and end-of-life discussions.

**Summary**

**What is already known?**

- Healthcare professionals record detailed conversations about a patient’s care during their end of life and although there is a drive to use standardised care pathways, real-world End of Life care is often very contextual and personalised. It is unknown to what extent this discussion of prognosis, ceilings of treatment or advanced directives occurs.

**What does this paper add?**

- This paper shows how computational AI approaches can measure how clincial language is used during End of Life and how this relates to prognosis and contextual meaning in an ecologically-valid manner.

## Introduction

Planning in advance for ‘End Of Life’ care is a complex and sensitive area of healthcare, and there is significant room for misunderstandings.^1^,^3^ Such discussions and advance decisions can be mishandled without personalised counselling as misperceptions may arise about what kinds of treatments are referred to.^4^ Phrases such as ‘ceiling of treatment’ and ‘treatment escalation plans’ attempt to clarify in more detail the context and the conversation of the different types of treatments being discussed. This has been supplemented by additional healthcare intervention approaches to improve standardisation of documentation of teams transcribing and transferring information relating to ceiling of treatment.^5 6^ As a result, there has been an expansion in the vocabulary around advanced directives and end-of-life care.

Traditional approaches using standardised forms or integrated care pathways to record such sensitive advance care plans have been extremely helpful in recording such complex personalised discussions between healthcare professionals with patients, families and carers.^7^ Many of such advance care plans are now captured in standardised electronic form templates often with details captured in typed free-text narrative. Often words and phrases in advance care plans have very specific technical meanings to a specialist which may not match intended meaning as interpreted by a non-specialist or a non-medical individual, for example: ‘not for cardiopulmonary resuscitation’ may get misinterpreted by an untrained reader to mean that the patient is having treatments withdrawn. Conventionally, studies in this domain have often used qualitative methodologies to disentangle this.^8^,^10^

To address this quantitative research gap, a computational linguistic approach was used to analyse large amounts of data using unsupervised algorithms to detect patterns in the use of words and phrases. This aims to give computers the ability to understand human language. This process is called natural language processing (NLP). The initial NLP approach used a data-driven technique called ‘Word2Vec’ to represent words from a large body of text in a multidimensional vector space (‘latent space’), based on the contextual use of surrounding words.^11^ With a sufficient body of text, these ‘word embeddings’ begin to cluster and words that cluster together often have similar meaning. These embeddings therefore follow the philosophical principle first coined by Ludwig Wittgenstein in 1953*“… the meaning of a word is its use in the language”.*^12^ This ecological data-driven approach has the advantage of also capturing jargon, acronyms and unconventional language that are being used in the real-world.

Using this data-driven approach in a large body of anonymised electronic clinical text at a large urban hospital in London, we analysed whether words or phrases (‘word embeddings’) discussing advance care planning and ceilings of treatment have similar semantic clusters. We also test whether there is any correlation of these ‘word embeddings’ with mortality, and how ‘word embeddings’ are abstracted by AI into ‘concept embeddings’.

## Methods

### Governance

Specific work on end-of-life care research was reviewed with expert patient input on a virtual committee with Caldicott Guardian oversight. Patient and public engagement was sought throughout this project with expert patients approving the projects as well as writing this article.

### Patient and public involvement

The project was proposed to a patient-led research committee and refined based on feedback. Subsequently, researchers performed the analysis and then produced initial results which was reviewed collectively with three expert patients and a manuscript written. Patient contributors wrote a patient-friendly abstract and are listed as coauthors in the manuscript.

### Study design and eligibility criteria

Cross-sectional retrospective study of all inpatient admissions of ≥1 day from October 2018 to October 2019. This corresponds to about 425 000 clinical episodes (see table 1).

**Table 1:** Word and phrase counts per inpatient were searched across all inpatient records along groups of similar semantics and linked to whether there was an associated date of death

	Key phrases showing up in documents from Octber 2018 to September 2019 (identical to ElasticSearch query)	Any inpatients with the phrase during time period in health record	Any inpatients with the phrase and death dates within 7 days	% of inpatients with phrase and death within 7 days	Relative risk versus annual control
Ceiling of Care Group	*“Treatment Escalation Plan”*	3181	55	1.7	2.16
	*“not for inotropes”*	20	<10	15.0	18.71
	*“not for hdu”*	39	<10	17.9	22.33
	*“currently for full”*	83	17	20.5	25.55
	*“ceiling of rx”* OR *“ceiling of care”* OR *“ceilings of care”* OR *“ceilings of treatment”* OR *“ceiling of treatment”*	1254	203	16.2	20.20
	“ceiling of care” OR “ceilings of care” OR “limit of care” OR “limits of care”	910	169	18.6	23.17
	“ceilings of treatment” OR “ceilings of treatment” OR “ceiling of rx”	431	54	12.5	15.63
	*“not for intubation”* OR *“not suitable for intubation”* OR *“not appropriate for intubation”*	184	51	27.7	34.58
	*“not for itu”* OR *“not for icu”* OR *“not suitable for itu”* OR *“not appropriate for itu”* OR *“not for escalation to itu”* OR *“not for critical care”*	284	99	34.9	43.49
	*“ward based ceiling of care”* OR *“'ward based care only”*	140	53	37.9	47.23
	*“not for escalation”* OR *“escalation beyond”*	193	75	38.9	48.48
	*“unsurvivable”*	59	34	57.6	71.89
End of Life Care Group	*“palliative treatments only”* OR *“palliative input”* OR *“palliative medications”* OR *“palliation”*	1165	390	33.5	41.76
	*“withdrawal of care”* OR *“withdrawal of treatment”* OR *“withdrawal of intensive”*	67	38	56.7	70.75
	*“terminal care”* OR *“end of life care”* OR *“eol care”* OR *“eolc”*	2138	1230	57.5	71.77
Control	None of the above phrases in either cluster	424 905	3406	0.8	–

Relative risk is derived from these absolute values.

### Data source and selection

The free-text corpus consists of ~18M documents spaning a 20-year period pooled from the CogStack platform at Kings College Hospital.^13^ CogStack harmonises data from the structured and unstructured components of the electronic health record. This includes all inpatient and outpatient documents. From the ~18M documents, we have removed form checklists and scanned documents of insufficient legibility, ending up with ~13M documents.

**Figure 1:**
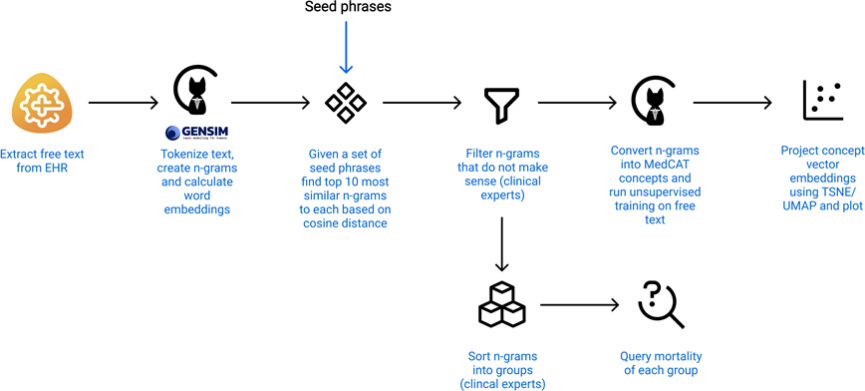
A flow diagram showing all steps taken from text extraction to plotting. EHR refers to the Electronic Health Record. This image is created by the authors.

### Unsupervised word and concept embeddings

The free-text corpus was first split into words, then put through a phraser which merged separate tokens into 2,3,4-Grams. For each N-Gram, a 300-dimensional vector embedding was calculated using *Word2Vec*^11^ with window_size=10, number_of_iterations=2 and minimum_word_frequency=10. All of this was done using MedCAT^14^ which internally relies on gensim.^15^ Given a set of root n-grams (“ceiling of care”, “withdrawal of care”, “limit of care”, “palliative treatments only”, “palliative care only”, “end of life care”, “liverpool care pathway”, “not for intubation”, “not for itu”, “not for critical care”), the top 10 most similar n-grams to each of the roots were collected based on the cosine distance between embeddings.

### Explanation of grouping of concept embeddings into meaning groups

After the top 10 n-grams were identified for each of the seed phrases, these were presented to the 3 healthcare professionals (one critical care physician, one palliative care physician and one neurologist) to group by meaning in human-determined clusters.

A CogStack ElasticSearch query was then performed for phrases within these clusters. An ElasticSearch query allows for a rapid search of the whole CogStack database (free text included) using keywords strings and filters (eg, keyword string = “ceiling of care”~5 AND [2018-10-01 TO 2019-09-30] AND filter = “inpatient”). These queries allow a degree of fuzzy querying with character inversions or mistypes as well as multiword proximity (eg, “family discussion”~5 searches for strings where the words “family”, “discussion” to occur within 5 words of each other so would include both “family discussion” and “discussion with the family”). Full details of this query syntax is available.^16^ The ElasticSearch query was used to generate total aggregate counts of unique inpatients with documents created in 2019 containing the phrases of interest. For each document containing a phrase of interest, we have also checked whether a date of death was recorded within 7 days of the date of the document. Seven days was chosen to limit the analyses to short-term prognostication. Dates of death were recorded based on the inpatient certification of death by doctor. As a control, all documents in the same time period without these phrases were used. The short time window provides confidence on accuracy on mortality data as any undercounting of outpatient mortality would not significantly impact the data.

### Visualisation of concept embeddings

All selected phrases were converted into MedCAT concepts. This simply means each phrase was assigned an ID and stored into a MedCAT concept database (CDB).^14^ The database holds pairs of phrase->ID. Each concept is an abstract entity rather than a concept linked to a health ontology. Once the database was created, we run the unsupervised training on the free text portion of KCH CogStack, excluding forms and bad scans (~13M documents). The unsupervised training calculates vector embeddings for concepts in the CDB, automatically dealing with spelling mistakes, metonyms and slight variations in the phrasing.

To visualise the relationship between the chosen concepts, t-distributed stochastic neighbour embedding (t-SNE) was used to reduce a high-dimensional vector (300 dimensions) into a two-dimensional space.^17^ In summary, t-SNE converts similarities between data points to joint probabilities and tries to minimise the Kullback-Leibler divergence between the joint probabilities of the low-dimensional embedding and the high-dimensional data. This plot ensures that word embeddings that are close in the high-dimensional space remain close in low-dimensional representation. An alternative dimensional reduction technique (Uniform Manifold Approximation and Projection^18^) was also tested and is available as a online supplemental file. The whole process from text extraction to plotting is shown in online supplemental figure N.

### Data availability

The source data will not be publicly available as the source data analysed is unstructured textual data, which carries risk of patient reidentification. The TSNE is available as a online supplemental HTML file.

### Code availability

The cogstack suite of tools (DrugPipeline,^19^ MedCAT^14^ and MedCATTrainer^20^) used for text extraction and NLP is available on https://github.com/CogStack under an open-source license (Apache V.2.0 license).

## Results

### Word embeddings

The seed n-gram’s “ceiling of care”, “withdrawal of care”, “limit of care”, “palliative treatments only”, “palliative care only”, “end of life care”, “liverpool care pathway”, “not for intubation”, “not for itu”, “not for critical care” were selected a priori by the healthcare team (see the Methods section), and the top 10 n-grams for each (up to four tokens) were consolidated, and the leading 40 n-grams are provided in table 2. A full list of phrases is available in online supplemental table 1. The leading 40 n-grams was chosen to reduce the mentions of irrelevant word fragments which are coassociated with end-of-life care but do not carry the intended semantic meaning, for example, partial prescriptions “morphine sulphate injection controlled”, “1 hour prn for agitation” or “to 5 mg subcutaneous”.

**Table 2:** List of n-grams from seed phrases (“ceiling of care”, “withdrawal of care”, “limit of care”, “palliative treatments only”, “palliative care only”, “end of life care”, “liverpool care pathway”, “not for intubation”, “not for itu”, “not for critical care”)

Not for cpr	Ceilings of care	Ward base	Family discussions
Ward based	Not for resuscitation	dnar not	Futility
Ward based ceiling of care	Ceiling of care	Escalation beyond	Organ donation
Escalation of care	Not for icu	dnacpr	Brainstem death
Not for itu	Not for niv	dnacpr and	Stem testing
Not for escalation	dnar and	dnar	Discussions with the family
Escalation and	Ceiling of treatment	Brainstem testing	Unsurvivable
Not for intubation	Resuscitation as	Withdrawal of intensive	Candidate for itu
Ward-based care only	Resusciation	Withdrawal of treatment	Ceilings of treatment
Not for resus	Palliation	Family discussion	Candidate for intubation

The acronyms identified from this approach were easily interpreted by an experienced clinician: “rx”=treatment; “eolc”=end of life care; “itu”=intensive therapy unit; “icu”=intensive care unit; “hdu”=high dependency unit; “dnacpr” for do not attempt cpr; “niv”=non-invasive ventilation.

### Relationship with outcome

The top n-grams above were then grouped together with phrases with similar meaning (poicelonyms), and then these text string groups were queried in the whole 2019 inpatient document dataset at Kings College Hospital to provide aggregated unique patients with those phrases (table 1). This is summarised in table 1 together with the numbers with and without recorded dates of death.

Phrases indicating “End of Life” and “Terminal” clearly had higher rates of mortality since it is implicit in their meaning, whereas terms referring to different limitations of LST had more intermediate prognosis. It is noteworthy that the preferred hospital protocol term to describe such discussions and plans in the hospital—“Treatment Escalation Plan” was extremely common (>3 k inpatients). However, this appeared to be used as a heading phrase, as it did not contain any semantic meaning on what the level of advance care was agreed. As a result, the 7-day mortality with “Treatment Escalation Plan” was extremely low. This suggests that these discussions are not foregone conclusions and that having such discussions does not carry an implicit implication of early mortality.

### Concept embeddings

**Figure 2:**
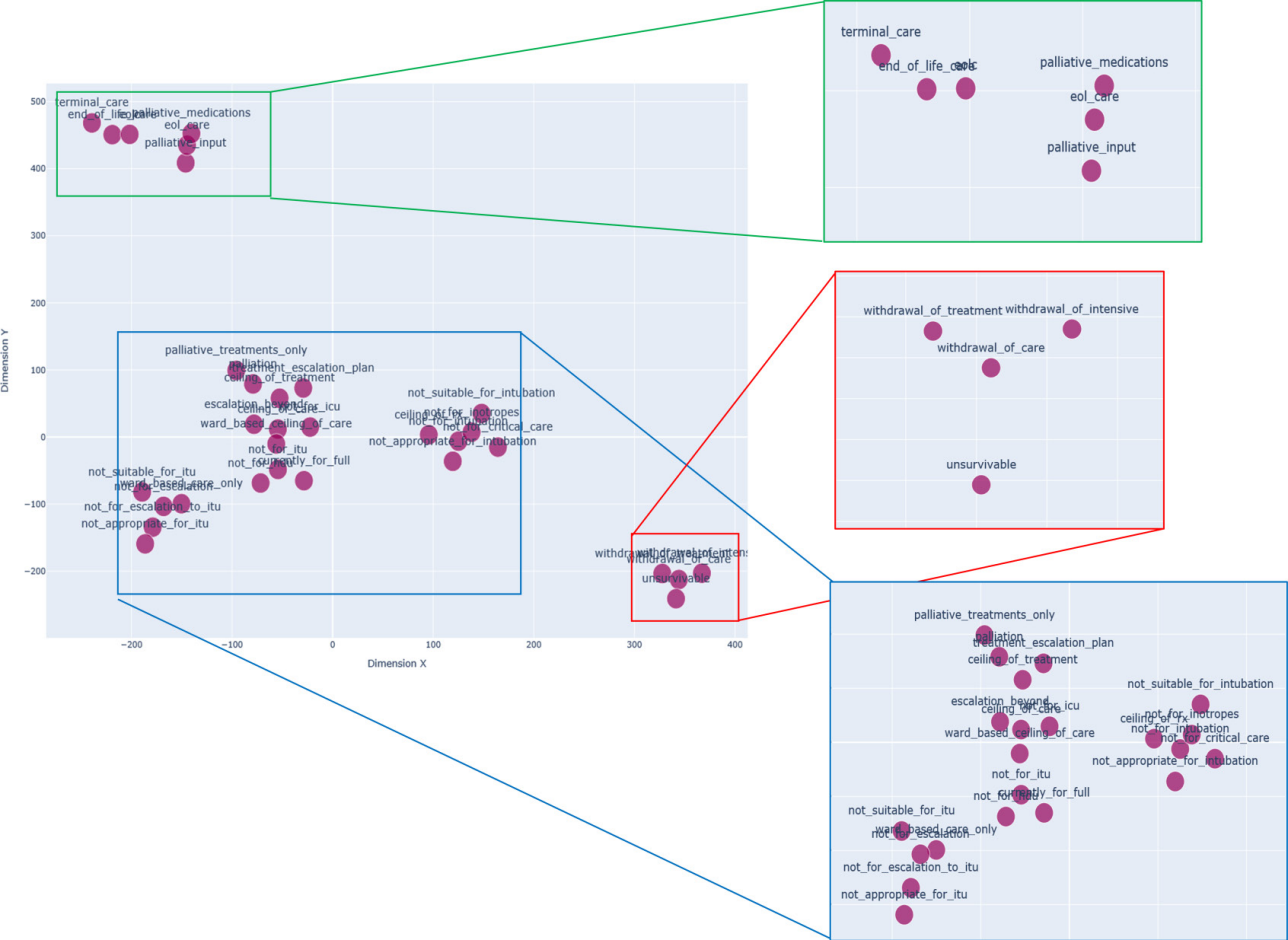
The clusters of concept embeddings on a t-distributed stochastic neighbour embedding (TSNE) plot in two-dimensions (X and Y). X and Y represent synthetic dimensions derived from the word embeddings, and is analogous to principle components in a principal component analysis. Regions of clustering are expanded for clarity with green–red clusters corresponding most similarly to End Of Life Care while blue cluster corresponding to Ceiling of Care. TSNE plot is available as dynamic figure in online supplemental HTML file. This image is created by the authors.

To correct for any misspellings and typographical errors, the word embeddings were converted to MedCAT concept embeddings and trained against the entire corpus. To visualise the semantic relationships between these concept embeddings, a t-distributed stochastic neighbour embedding (t-SNE) was used to reduce a high-dimensional vector (300 dimensions) into a two dimensional in figure 1.^17^ There are three broad groups which only partially follow the clinical groupings used in table 1. Of note, the regions outlined in green and red in this two-dimensional semantic space in figure 2 correspond to the ‘End of Life’ grouping in table 1 where the outcomes are poorest. Less discrete clusters in the blue regions with n-grams of overlapping outcomes describing limits of appropriate interventions similar in meaning to the Ceiling of Care group.

## Discussion

We present the first quantitative NLP evaluation of the language used in real-world discussions about ceiling of treatments and End Of Life care.

The principal finding is that there is substantial varied real-world language describing advance care planning ranging from specific interventions to terminal prognostication by clinical teams, and these captured implicit and inferred poor prognosis. This study also showed that unsupervised word-embedding machine learing techniques (Word2Vec and MedCAT) were able to produce clusters of phrases which reflect phrases of similar meaning using dimensionality reduction techniques.

This study therefore has an inverted design to a previous Sentiment Analysis study of nursing notes from the The Multiparameter Intelligent Monitoring in Intensive Care (MIMIC-III) public intensive care unit dataset which found a relationship between such ‘sentiment’ with survival;^21^ the ‘sentiment’ was calculated using a rules-based semantic analysis tool (TextBlob^22^) designed for generic non-clinical text which assigns a positive or negative ‘sentiment’ score to a piece of text based on the adjectives, verbs and adverbs used in the text.^23 24^ In the current study, both an *a priori* approach and an unsupervised clustering approach were used showing clear associations with the ‘ground truth’ of mortality. The derivation of ‘sentiment’ on prognosis from real-world clinical text also makes this much more ecological rather than using rule-based text analysis designed for non-clinical uses.

One significant limitation is that this study did not explore temporal trends in prognosis or embeddings. The scope of this study was the ceiling of treatments towards the end of life and so the focus was very much on the discussions and words used very near the end of life (ie, within the next 7 days). This narrows the vocabulary for prognosis without introducing noise around the vocabulary of tenses and accuracy of time-course prognostication. Another limitation is the lack of distinction between the different types of ceiling of treatment scenarios; it is likely a ceiling of treatment discussion about an elderly disabled patient is substantially different to that of a young patient with a terminal illness or a sudden traumatic event. Both aspects could be improved on with an expanding corpus as well as exploring the temporal relationship with medical and palliative interventions.

During this study, typographical errors and metonymic variations on free-text data entry was frequently detected, requiring an addition of a concept embedding approach using MedCAT. These variations in typing suggest that clinicians do not simply copy-and-paste templated thoughts for a very ill patient but instead provide contextualised care to the individual (with manually composed typing) even in an era of increasing standardisation of care pathways.

In summary, our study maps out how clinical language is used to describe ‘End Of Life’ discussions in real-world scenarios as well as to produce syntactic phrase or word clusters that capture information on short-term prognosis and supplements qualitative approaches. Future work could explore the use of language in different professional groups or explore the temporality of interventions before and after such discussions.

**Patient-friendly summary by expert patients: Sherry Charing, Alan Quarterman, Harold Parkes**

Discussions between doctors, patients and family in deciding what is the appropriate maximum treatment a specific patient should have based on their clinical condition is complex. Discussions, often involving expressions regarding ‘End Of Life’ care, are used to describe the maximum invasive treatments a patient should have or would want. There are a range of expressions used, many with overlapping meanings which can be confusing, not only for the patient and family, but also for doctors reading the patient’s clinical notes. In this study, a computational approach using artificial intelligence (AI) to read clinical patient notes was carried out by looking at thousands of patient records from a large urban hospital. Expressions that doctors use to describe these discussions were analysed to show the associations of particular words and phrases in relation to mortality. Using a computer analysis for this study, it was possible to quantify the use of these expressions and their relation to the ‘End Of Life’. Through this AI-based approach, real-world use of phrases and language relating ‘End Of Life’ can be analysed to understand how doctors and patients are communicating, and about any possible misunderstandings of language.

## Supplementary material

10.1136/bmjhci-2021-100464 online supplemental file 1

10.1136/bmjhci-2021-100464 online supplemental file 2

## Data Availability

No data are available.
