# Supplementary figures and images for: Natural language word embeddings as a glimpse into healthcare language and associated mortality surrounding end of life
Source: BMJ Health Care Inform. 2021 Oct 27;28(1):e100464. doi: 10.1136/bmjhci-2021-100464 (PMC8557276; doi:10.1136/bmjhci-2021-100464)

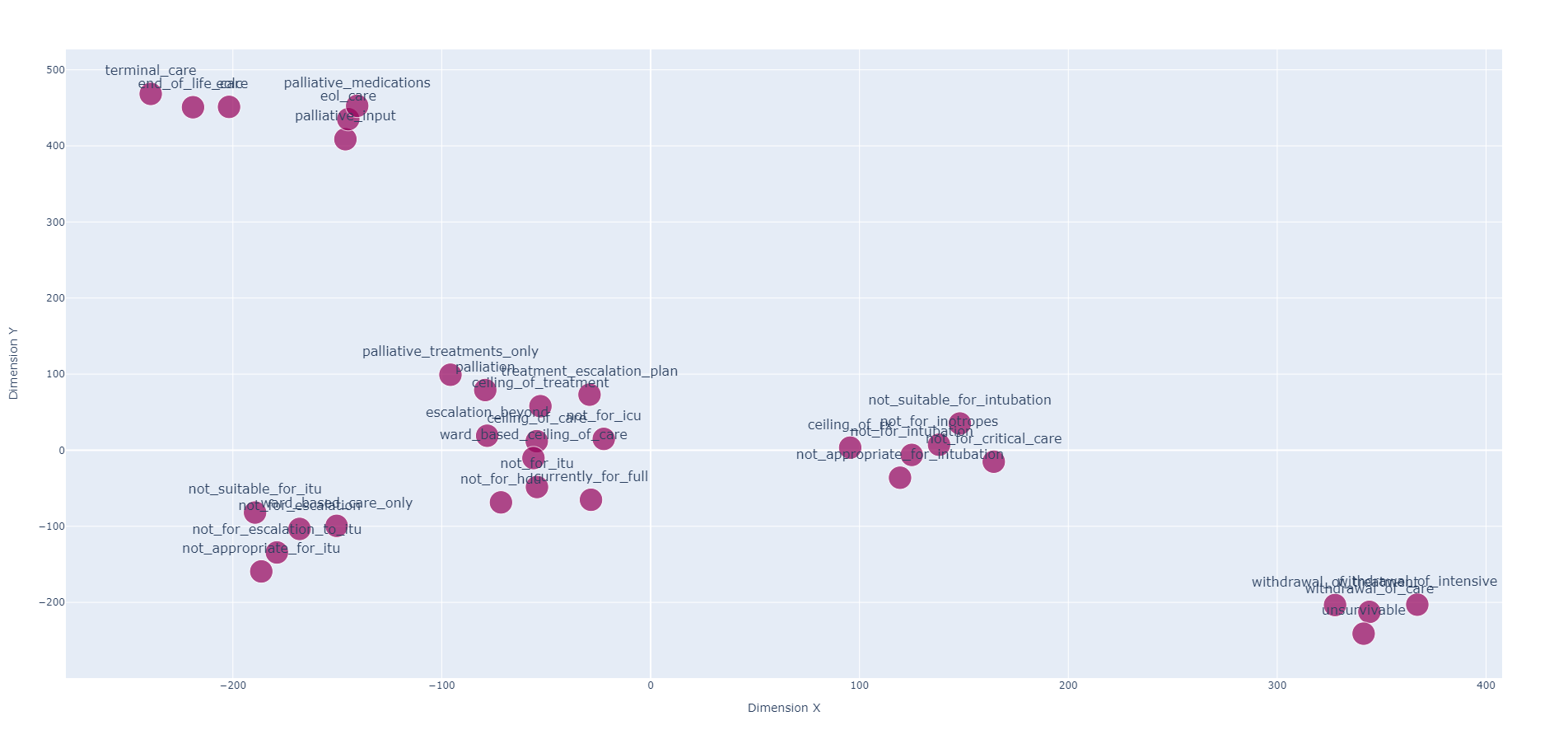

Supplement: online supplemental file 1 [file bmjhci-2021-100464supp001.png]
