# Supplementary material for: Natural language word embeddings as a glimpse into healthcare language and associated mortality surrounding end of life
Source: BMJ Health Care Inform. 2021 Oct 27;28(1):e100464. doi: 10.1136/bmjhci-2021-100464 (PMC8557276; doi:10.1136/bmjhci-2021-100464)
Supplement: online supplemental file 2 [file bmjhci-2021-100464supp002.docx]

**Supplementary Table 1: All n-grams without filtering**

| **Seed Phrase:**  **"Ceiling of Treatment"** | **Alternative Seed Phrase: "DNAR"** | **Alternative Seed Phrase:**  **"End of Life"** |
| --- | --- | --- |
| not for cpr | not_for_resus | 'eol_medications' |
| ward based | not_for_cpr | 'end_of_life_care' |
| ward based ceiling of care | dnacpr | 'comfort_care' |
| escalation of care | ceiling_of_care | 'eol_meds' |
| not for itu | dnar_and | 'eolc' |
| not for escalation | dnar | 'not_for_cpr' |
| escalation and | ward_based_ceiling_of_care | 'palliative_medications' |
| not for intubation | not_for_itu | 'eol_care' |
| ward based care only | ward_based | 'prn_eol' |
| active ward | dnacpr_and | 'eolc_medications' |
| not for resus | not_for_escalation | 'active_ward' |
| ceilings of care | ceilings_of_care | 'palliative_meds' |
| not for resuscitation | not_for_resuscitation | 'ward_based_ceiling_of_care' |
| ceiling of care | dnar_l1 | 'ward_based' |
| based management | ceiling_of_treatment | 'anticipatory_medications' |
| not for icu | escalation_of_care | 'eol_prn' |
| not for niv | community_dnar | 'not_for_itu' |
| dnar and | community_dnacpr | 'anticipatory_meds' |
| ceiling of treatment | not_for_intubation | 'palliation' |
| resuscitation as | dnr | 'palliative_input' |
| cpr or | eol | 'ward_based_care_only' |
| resusciation | and_dnar | 'ward_base' |
| palliation | dnar_in | 'palliative_referral' |
| ward base | ward_based_care_only | 'end_of_life_care_and' |
| dnar not | active_ward | 'dnar_and' |
| escalation beyond | dnar_not | 'ceiling_of_care' |
| dnacpr | escalation_and | 'not_for_resus' |
| dnacpr and | dnacpr_in | 'eol' |
| dnar | dnar_in_place | 'actively_dying' |
| brainstem testing | ward_base | 'palliation_and' |
| withdrawal of intensive | comfort_care | 'not_for_escalation' |
| withdrawal of treatment | dna_cpr | 'hospice_transfer' |
| family discussion | nfr | 'prn_eolc' |
| family discussions | dnr_in | 'hospice_bed' |
| futility | eol_care | 'based_management' |
| organ donation | care_dnar | 'palliative_care_input_and' |
| brainstem death | dnar_decision | 'home_for_end_of_life_care' |
| itu consultant | not_for_niv | 'plan_eol' |
| neurological prognosis | ceilings_of_treatment | 'dnar_not' |
| stem testing | dnar_signed | 'not_for_intubation' |
| discussions with the family | dnr_form | 'palliative_review' |
| unsurvivable | dnar_with | 'care_input' |
| candidate for itu | dnar_l2 | 'palliative_care_input' |
| resusitation | currently_for_full | 'ongoing_palliative' |
| rescusitation | dnacpr_l1 | 'palliative_care_team_review' |
| candidate for intubation | dnacpr_level | 'palliative_care_team_input' |
| dnacpr status | and_dnacpr | 'to_hospice' |
| ceilings of treatment | dnar_status | 'nh_placement' |
| escalation plans | resus_dnar | 'track_funding' |
| cpr status | level_care | 'escalation_of_care' |
| escalation level | dnar_as | 'life_care' |
| eolc | community_dnr | 'on_eol' |
| eol care | dnacpr_status | 'end_of_life_care_at' |
| comfort care | cpr_status | 'eol_medication' |
| end of life care and | previous_dnar | 'hospice_placement' |
| palliative medications | eolc | 'refer_to_palliative_care' |
| palliative input | palliative_input | 'palliative_approach' |
| hospice placement | re_dnar | 'prn_palliative' |
| eol medications | end_of_life_care | 'transfer_to_st_christopher' |
| eol meds | palliation | 'hospice_referral' |
| plan eol | eol_meds | 'palliative_and' |
| terminal care | eol_medications | 'ceilings_of_treatment' |
| palliation and | actively_dying | 'end_of_life_care_medications' |
| 1 hour prn for agitation | nh_resident | 'dnr_not' |
| controlled drug 2 5 | in_nh | 'nfr' |
| secretions liverpool care pathway | background_dementia | 'dnacpr' |
| to 5 mg subcutaneous | home_resident | 'eolc_prn' |
| 1 to 2 mg | dementia_bedbound | 'ceilings_of_care' |
| liverpool care pathway verified | bg_dementia | 'escalation_plans' |
| lactulose solution | dnar_level | 'ceiling_of_treatment' |
| 5 mg subcutaneous every | family_updated | 'continue_palliative' |
| midazolam injection | resusitation | 'seen_by_palliative_team' |
| morphine sulphate injection controlled | dnar_from | 'eol_pathway' |
| every 1 hour prn | dols_in | 'not_for_resuscitation' |
| sulphate injection | advanced_dementia | 'a_nursing_home_placement' |
| prn for agitation | nfr_and | 'end_of_life_care_i' |
| control of upper respiratory tract | dnar_but | 'and_end_of_life_care' |
| drug 2 5 to | dnar_cpr | 'prn_medications' |
| escalation to | not_for_icu | 'palliative_management' |
| or intubation | based_care | 'keep_comfortable' |
| dnar ward | and_escalation | 'care_meds' |
| currently for full | dementia_dnar | 'hospice_admission' |
| plan dnar | alzheimers_dementia | 'place_of_care' |
| with icu | alzheimers | 'st_christopher_s' |
| not for hdu | palliative_management | 'care_funding' |
| beyond ward | palliation_and | 'and_palliation' |
| maximal ward | palliative_approach | 'best_supportive_care_and' |
| ceiling of rx | dnr_level | 'a_palliative' |
| in event of cardiac arrest | 3_dnar | 'that_palliative' |
| not for inotropes | escalation_level | 'terminal_care' |
| escalation or | 1_dnar | 'preferred_place_of_care_is' |
| ceiling of care | dnacpr_with | 'discharge_to_a_nursing' |
| withdrawal of care | with_dnar | 'end_of_life_care_in' |
| limit of care | discussed_dnacpr | 'community_palliative_care' |
| limits of care | about_resuscitation | 'chaplaincy_support' |
| palliative treatments only | has_dnar | 'chaplaincy_referral' |
| end of life care | peace_document | 'consider_hospice' |
| liverpool care pathway | nfr_not | 'to_discuss_with_family' |
| not for intubation | based_management | 'injectable_medications' |
| not suitable for intubation | dnr_not | 'anticipatory_medication' |
